# Patterns in Patient Encounters and Emergency Department Capacity in California, 2011-2021

**DOI:** 10.1001/jamanetworkopen.2023.19438

**Published:** 2023-06-22

**Authors:** Renee Y. Hsia, Stefany Zagorov, Nandita Sarkar, Michael T. Savides, Madeline Feldmeier, Newton Addo

**Affiliations:** 1 Department of Emergency Medicine, University of California, San Francisco; 2 Philip R. Lee Institute for Health Policy Studies, University of California, San Francisco; 3 National Bureau of Economic Research, Cambridge, Massachusetts

## Abstract

**Importance:**

The health care system has undergone major changes in the past decade, and emergency department (ED) crowding has worsened over time; however, the most recent patterns in ED capacity and use in California have yet to be studied.

**Objective:**

To analyze patterns in ED capacity and utilization in California hospitals from 2011 to 2021.

**Design, Setting, and Participants:**

This retrospective cohort study used data from the California Department of Health Care Access and Information and the US Census Bureau to analyze ED facility characteristics from more than 400 general acute care hospitals with more than 320 EDs in California as well as patients who presented to those EDs between January 1, 2011, and December 31, 2021.

**Main Outcomes and Measures:**

Linear patterns (measured by percentage change) in total annual ED capacity (volume of hospital beds, EDs, ED treatment stations, and trauma centers) and ED use (ED visits by disposition and acuity) were assessed as primary outcomes. Patterns in ambulance diversion hours and the number of patients who left the ED without being seen were also examined as secondary outcomes. Visit acuity was categorized into 5 levels by increasing severity (minor, low to moderate, moderate, severe without threat, and severe with threat) based on California Department of Health Care Access and Information descriptions corresponding to *Current Procedural Terminology* codes.

**Results:**

In the prepandemic period (2011-2019), the total population of California increased from 37 638 369 to 39 512 223 (5.0%; 95% CI, 4.1%-5.8%), then decreased to 39 237 836 in 2021 (0.7%; 95% CI, −3.9% to 2.5%). Over the entire study period (2011-2021), the total California population increased by 4.2% (95% CI, 3.3%-5.2%). From 2011 to 2019, the annual number of ED visits increased from 12 054 885 to 14 876 653 (23.4%; 95% CI, 20.0%-26.8%) before decreasing to 12 944 692 in 2021 (−13.0%; 95% CI, −33.1% to 7.1%); from 2011 to 2021, total ED visits increased by 7.4% (95% CI, 5.6%-9.1%). From 2011 to 2021, the total number of EDs decreased from 339 to 326 (−3.8%; 95% CI, −4.4% to −3.2%) and the total number of hospital beds decreased from 75 940 to 74 052 (−2.5%; 95% CI, −3.3% to −1.6%), while the number of ED treatment stations in these fewer EDs increased from 7159 to 8667 (21.1%; 95% CI, 19.7%-22.4%). The number of visits rated as severe with threat also increased, from 2 011 637 in 2011 to 3 375 539 in 2021 (67.8%; 95% CI, 59.7%-75.9%), while visits rated as minor decreased from 913 712 to 336 071 (−63.2%; 95% CI, −75.2% to −51.2%) over the same period.

**Conclusions and Relevance:**

In this cohort study, multiple measures of ED capacity did not proportionally increase with the increasing demand for services; however, the COVID-19 pandemic appears to have substantially affected some of these patterns. These findings may be helpful to policy makers and health care stakeholders when planning resource allocation of limited health care resources.

## Key Points

**Question:**

How have emergency department (ED) capacity and use changed in California since 2011, and has the supply of acute care resources kept up with the demand for ED care?

**Findings:**

In this cohort study of ED data from more than 400 general acute care hospitals in California between 2011 and 2021, the number of EDs decreased by 3.8% and the number of hospital beds decreased by 2.5%; the number of treatment stations increased by 21.1%, but these stations were concentrated in a smaller number of EDs. From 2011 to 2019, the total number of ED visits increased by 23.4%, and visits rated as severe with threat (*Current Procedural Terminology* code 99285) increased by 67.8% over the entire study period.

**Meaning:**

These findings suggest that, although some mixed results were present, ED capacity has not proportionally expanded with the increasing California population and demand for emergency services, offering 1 potential explanation for increases in ED crowding.

## Teaser

This cohort study uses data from the California Department of Health Care Access and Information and the US Census Bureau to analyze patterns in emergency department capacity and use in California hospitals from 2011 to 2021.

## Introduction

Emergency departments (EDs) play an increasingly important role in the US health care system. From 2003 to 2009, hospital admissions from the ED increased by 17%, while admissions from physicians’ offices and clinics decreased by 10%.^1^ By 2018, EDs accounted for 70% of all US hospital admissions^2^ compared with 2009, when EDs only accounted for approximately 50% of admissions.^1^ Under the Emergency Medical Treatment and Labor Act,^3^ EDs are required to stabilize all patients with emergent conditions, regardless of the patient’s ability to pay. In this way, EDs are distinct in that they provide a safety net of uncompensated care.^4^ Furthermore, by handling overflow and after-hours care, EDs have increasingly supported primary care practices. In fact, the majority of ambulatory patients use the ED because they do not have any other timely alternative for care.^1,4^

Given this increased burden on EDs, ensuring a sufficient supply of ED resources is important, particularly for California, which ranked ninth in the nation in 2022 for states with the longest ED waiting times, with a median waiting time of 164 minutes.^5^ Crowding in the ED is a substantial concern because it has been associated with increased mortality,^6,7,8,9^ longer lengths of stay,^10,11^ and clinician error.^12,13^

Given the growing role of EDs in the US health care system, we sought to examine patterns in ED supply and use in general acute care (GAC) hospitals in California from 2011 to 2021. Using state-level data, we examined changes in the supply of EDs, trauma centers (TCs), treatment stations, and hospital beds relative to use (ED visits by disposition and acuity). By comparing ED supply with the demand for care, we aimed to assess which populations and areas of the health care system exhibited a growing need for ED resources and where supply was meeting demand. Our analysis of ED data over this period also included the COVID-19 pandemic years of 2020 and 2021, allowing us to evaluate the exacerbation or stagnation of previous secular trends associated with the pandemic. We hypothesized that from 2011 to 2021, ED use would grow more quickly than ED supply and that ED visit rates and visits per treatment station would increase, especially among patients with high-acuity visits.

## Methods

### Study Design and Data Sources

The institutional review board of the University of California, San Francisco approved this study with a waiver of informed consent because of the use of deidentified data that did not constitute human participant research, in accordance with the university’s Human Research Protection Program guidelines^14^ and the Common Rule (45 CFR §46).^15^ This study followed the Strengthening the Reporting of Observational Studies in Epidemiology (STROBE) reporting guideline for cohort studies.

We analyzed public files on annual hospital and ED use from 2011 to 2021 from the California Department of Health Care Access and Information, which conducts annual surveys of all hospitals and hospital systems in California.^16,17^ California population estimates were determined using annual data from the US Census Bureau.^18,19^

### Inclusion Criteria and Variable Definitions

Our study included all GAC facilities in California with EDs that were not closed at the end of the year and were operational at any time during the year. Primary outcomes were linear patterns in total annual ED capacity and ED use. We included all ED visits reported to the state; ED visits were classified as inpatient or admitted if the visit resulted in a hospital admission and outpatient or discharged if the visit resulted in a discharge from the ED. Visit acuity was categorized into 5 levels of increasing severity using *Current Procedural Terminology* (*CPT*) codes. These levels are described by the California Department of Health Care Access and Information as minor (*CPT* code 99281), low to moderate (*CPT* code 99282), moderate (*CPT* code 99283), severe without threat (*CPT* code 99284), and severe with threat (*CPT* code 99285). Categorization of TCs was based on Emergency Medical Services Authority data.^20^ Capacity measures refer to the data pertaining to hospitals, hospital beds, EDs, TCs, and ED treatment centers. Measures of use refer to the data pertaining to ED visits, admissions, discharges, and visit acuity. Ambulance diversion hours and patients who left without being seen were secondary outcomes.

### Statistical Analysis

We assessed patterns for each of the following measures: annual volume of GAC hospitals, hospital beds, TCs, EDs, ED treatment stations, total ED visits, visits resulting in admission or discharge, visits by acuity, ambulance diversion hours, and patients who left without being seen. We calculated the percentage change for each measure from January 1, 2011, to December 31, 2021, with 2011 as the reference year. Figures for ED visit rates per 1000 people, hospital beds per 1 million people, ED visits per treatment station, and treatment stations per 1 million people were also calculated. Linear patterns were assessed with R statistical software, version 4.1 (R Foundation for Statistical Computing), using linear regression models over the 3 periods (2011-2019, 2019-2021, and 2011-2021). The threshold for statistical significance was 2-tailed *P* < .05.

## Results

### Hospital Characteristics

**Figure 1.**
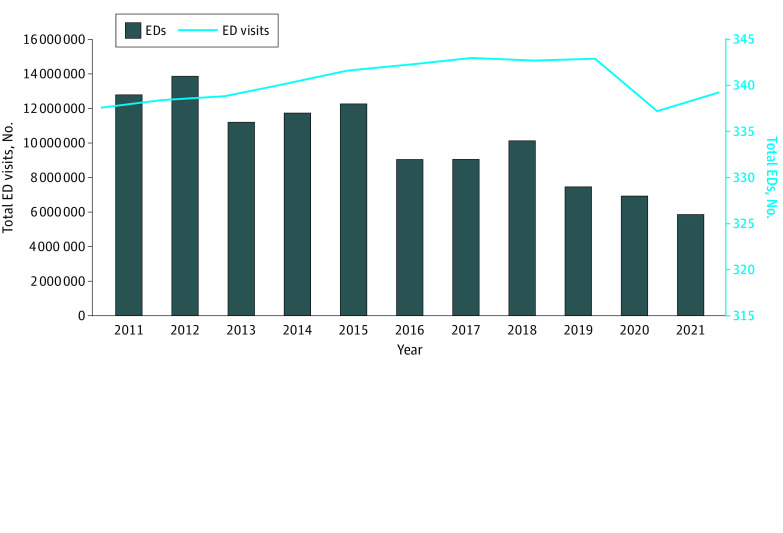
Emergency Department (ED) Visits and Total EDs in California, 2011-2021

Among all GAC hospitals in California from 2011 to 2021, the total number of hospitals decreased from 434 to 407 (−6.2%; 95% CI, −6.9% to −5.5%; *P* < .001) (Table 1). There were 13 fewer hospitals with an ED (from 339 in 2011 to 326 in 2021; −3.8%; 95% CI, −4.4% to −3.2%; *P* < .001) (Figure 1) and 14 fewer hospitals without an ED (from 95 in 2011 to 81 in 2021; −14.7%; 95% CI, −17.4% to −12.1%; *P* < .001). The number of government-owned hospitals decreased from 68 in 2011 to 60 in 2021 (−11.8%; 95% CI, −13.5% to −10.0%; *P* < .001), while there was no significant change in the number of for-profit hospitals (67 in both 2011 and 2021; 0%; 95% CI, −3.2 to 3.2; *P* = .92) or not-for-profit hospitals (204 in 2011 vs 199 in 2021; −2.5%; −3.9 to −1.0; *P* = .09).

**Table 1.** Emergency Department Facilities, Hospital Capacity, and Ownership of Hospitals With an Emergency Department

Characteristic	Year											% Change (95% CI)	*P* value
	2011	2012	2013	2014	2015	2016	2017	2018	2019	2020	2021		
Facilities													
Total GAC hospitals	434	433	428	433	427	423	418	420	413	415	407	−6.2 (−6.9 to −5.5)	<.001
GAC hospitals without an ED	95	92	92	96	89	91	86	86	84	87	81	−14.7 (−17.4 to −12.1)	<.001
GAC hospitals with an ED	339	341	336	337	338	332	332	334	329	328	326	−3.8 (−4.4 to −3.2)	<.001
ED level of service													
Comprehensive	9	9	9	9	8	9	9	9	11	11	11	22.2 (13.0 to 31.4)	.01
Basic	293	296	293	293	296	292	293	291	289	287	286	−2.4 (−3.1 to 1.7)	.001
Standby	37	36	34	35	34	31	30	34	29	30	29	−21.6 (−26.0 to −17.3)	<.001
EDs by No. of TCs													
Adult													
No TC	274	273	268	264	266	260	257	259	255	254	252	−8.0 (−8.8 to −6.9)	<.001
Total TCs	65	68	68	73	72	72	75	75	74	74	74	13.8 (10.7 to 17.0)	<.001
Level I	13	13	13	13	12	13	13	15	15	14	14	7.7 (1.3 to 14.1)	.03
Level II	32	33	33	35	37	36	37	35	35	36	36	12.5 (8.1 to 16.9)	.02
Level III	11	13	13	14	13	13	14	14	13	13	13	18.2 (10.1 to 26.2)	.22
Level IV	9	9	9	11	10	10	11	11	11	11	11	22.2 (15.8 to 28.7)	.001
Pediatric													
No TC	329	327	322	322	322	315	315	317	312	312	310	−5.8 (−6.4 to −5.1)	<.001
Total TCs	10	14	14	15	16	17	17	17	17	16	16	60.0 (43.5 to 76.5)	.009
Level I	5	5	5	5	5	5	5	7	7	6	6	20.0 (6.3 to 33.7)	.02
Level II	5	9	9	10	11	12	12	10	10	10	10	100.0 (62.6 to 137.4)	.10
Hospital capacity													
Total beds	75 940	76 996	76 339	75 658	75 404	75 332	75 967	73 722	74 580	74 114	74 052	−2.5 (−3.3 to −1.6)	<.001
Beds per million people	2018	2029	1995	1960	1938	1923	1930	1868	1888	1876	1887	−6.5 (−7.6 to −5.3)	<.001
Total treatment stations	7159	7382	7555	7743	7878	7859	8056	8152	8362	8723	8667	21.1 (19.7 to 22.4)	<.001
ED visits per treatment station	1684	1694	1684	1735	1802	1853	1853	1813	1779	1360	1494	−11.3 (−21.3 to −1.3)	.29
Treatment stations per million people	190	195	197	201	202	201	205	207	212	221	221	16.1 (14.5 to 17.8)	<.001
Hospital ownership													
Government	68	68	66	66	67	64	61	62	61	61	60	−11.8 (−13.5 to −10.0)	<.001
Not for profit	204	207	207	208	209	206	208	208	204	202	199	−2.5 (−3.9 to −1.0)	.09
For profit	67	66	63	63	62	62	63	64	64	65	67	0 (−3.2 to 3.2)	.92

Abbreviations: ED, emergency department; GAC, general acute care; TC, trauma center.

### Hospitals With TCs

From 2011 to 2021, the number of GAC hospitals with TCs increased from 65 to 74 (13.8%; 95% CI, 10.7%-17.0%; *P* < .001), and the number of pediatric TCs increased from 10 to 16 (60.0%; 95% CI, 43.5%-76.5%; *P* = .009) (Table 1). Over the same period, the number of hospitals without a TC decreased slightly from 274 to 252 (−8.0%; 95% CI, −8.8% to −6.9%; *P* < .001), and the number of hospitals without a pediatric TC decreased from 329 to 310 (−5.8%; 95% CI, −6.4% to −5.1%; *P* < .001). The number of TCs at each of the 4 levels increased, with the largest change in level IV TCs (from 9 in 2011 to 11 in 2021; 22.2%; 95% CI, 15.8%-28.7%; *P* = .001). There was 1 additional level I pediatric TC over the study period (from 5 in 2011 to 6 in 2021; 20.0%; 95% CI, 6.3%-33.7%; *P* = .02), and the number of level II centers doubled (from 5 in 2011 to 10 in 2021; 100.0%; 95% CI, 62.6%-137.4%; *P* = .10).

### ED Capacity

Between 2011 and 2021, the number of hospital beds decreased from 75 940 to 74 052 (−2.5%; 95% CI, −3.3% to −1.6%; *P* < .001), while the number of ED treatment stations increased from 7159 to 8667 (21.1%; 95% CI, 19.7%-22.4%; *P* < .001) (Table 1). Over the same period, hospital beds per 1 million people decreased from 2018 to 1887 (−6.5%; 95% CI, −7.6% to −5.3%; *P* < .001), and ED treatment stations per 1 million people increased from 190 to 221 (16.1%; 95% CI, 14.5%-17.8%; *P* < .001). The number of ED visits per treatment station decreased by 11.3% (95% CI, −21.3% to −1.3%; *P* = .29) over the entire study period. Although this decrease in ED visits per treatment station was not significant, 2 distinct patterns were observed: visits per treatment station increased from 1684 in 2011 to 1779 in 2019 (5.7%; 95% CI, 2.4%-8.9%; *P* = .01), then decreased to 1494 in 2021 (−16.0%; 95% CI, −38.8% to 6.7%; *P* = .54).

Furthermore, the distribution of EDs across the 3 levels of service (standby, basic, or comprehensive) changed significantly. From 2011 to 2021, the volume of EDs with comprehensive licensing increased from 9 to 11 (22.2%; 95% CI, 13.0%-31.4%; *P* = .01), while the number of basic EDs decreased from 293 to 286 (−2.4%; 95% CI, −3.1% to 1.7%; *P* = .001), and the number of standby EDs decreased from 37 to 29 (−21.6%; 95% CI, −26.0% to −17.3%; *P* < .001) (Table 1).

### Total California Population and ED Visits

In the prepandemic period (2011-2019), the California population increased from 37 638 369 to 39 512 223 (5.0%; 95% CI, 4.1%-5.8%; *P* < .001), then decreased to 39 237 836 in 2021 (−0.7%; 95% CI, −3.9% to 2.5%; *P* = .31). Over the entire study period, the total California population increased by 4.2% (95% CI, 3.3%-5.2%; *P* < .001) (Table 2).

**Table 2.** Descriptive Characteristics of Emergency Department Visits

Characteristic	Year^a^											% Change (95% CI)	*P* value
	2011	2012	2013	2014	2015	2016	2017	2018	2019	2020	2021		
Total population	37 638 369	37 948 800	38 260 787	38 596 972	38 918 045	39 167 117	39 358 497	39 461 588	39 512 223	39 499 738	39 237 836	4.2 (3.3 to 5.2)	<.001
Total ED visits	12 054 885	12 505 718	12 722 085	13 436 083	14 198 173	14 560 356	14 928 933	14 781 546	14 876 653	11 860 597	12 944 692	7.4 (5.6 to 9.1)	.37
Discharged ED visits^b^	10 133 025	10 679 293	10 901 245	11 590 545	12 339 530	12 643 396	12 996 528	12 884 768	12 943 549	10 021 952	10 963 240	8.2 (−4.5 to 21.0)	.67
Admitted ED visits^b^	1 813 434	1 728 142	1 718 109	1 771 643	1 785 124	1 845 010	1 839 514	1 803 747	1 855 365	1 768 364	1 917 196	5.7 (2.8 to 8.7)	.40
ED visits per 1000 people	320	330	333	348	365	372	379	375	377	300	330	3.0 (−6.7 to 12.7)	.04
Visits by severity^c^													
Minor	913 712	796 173	800 986	801 400	808 106	776 707	733 601	703 543	450 142	316 722	336 071	−63.2 (−75.2 to −51.2)	<.001
Low to moderate	2 032 758	2 014 199	2 045 532	2 065 657	2 170 099	2 262 902	2 103 459	2 058 891	1 826 148	1 310 505	1 520 222	−25.2 (−37.8 to −12.6)	.03
Moderate	4 187 242	4 492 906	4 684 705	4 884 701	5 191 179	5 315 877	5 433 919	5 074 777	4 749 307	3 643 358	3 848 953	−8.1 (24.1 to 7.9)	.52
Severe without threat	2 801 110	3 063 915	3 101 587	3 349 631	3 550 280	3 595 306	3 808 953	3 793 604	4 399 317	3 434 760	3 799 651	35.6 (29.8 to 41.5)	.004
Severe with threat	2 011 637	2 040 242	1 986 544	2 260 799	2 404 990	2 537 614	2 756 110	3 057 700	3 374 000	3 084 971	3 375 539	67.8 (59.7 to 75.9)	<.001
Missing	108 426	98 283	102 731	73 895	73 519	71 950	92 891	93 031	77 739	70 281	64 256	−40.7 (−52.4 to −29.1)	.02
Left without being seen, visits	303 560	314 029	309 493	310 888	363 152	326 444	321 833	299 275	331 401	270 826	315 023	3.8 (−4.8 to 12.4)	.66
Ambulance diversion, h	83 558	82 792	60 970	72 606	96 443	94 654	82 498	72 379	77 509	93 311	162 217	94.1 (62.9 to 125.4)	.09

Abbreviation: ED, emergency department.

^a^ All data reported in this table (other than total population data) were obtained from the California Department of Health Care Access and Information annual hospital use files. Total population data were obtained from the US Census Bureau.

^b^ Discharged and admitted ED visits were calculated by summing the total discharged and admitted visits by severity for each year. The sum of all visits by severity varied less than 1% each year from the total population reported in the corresponding use file.

^c^ Severity levels for visit acuity were based on California Department of Health Care Access and Information^16,17^ descriptions corresponding to *Current Procedural Terminology* codes, with minor corresponding to code 99281, low to moderate corresponding to code 99282, moderate corresponding to code 99283, severe without threat corresponding to code 99284, and severe with threat corresponding to code 99285.

Before the pandemic, total annual ED visits in California increased from 12 054 885 in 2011 to 14 876 653 in 2019 (23.4%; 95% CI, 20.0%-26.8%; *P* < .001) before decreasing to 12 944 692 in 2021 (−13.0%; 95% CI, −33.1% to 7.1%; *P* = .56). From 2011 to 2021, ED visits increased by 7.4% (95% CI, 5.6%-9.1%; *P* = .37), but this pattern was not significant (Figure 1).

When adjusting for population growth, the ED visit rate from 2011 to 2019 increased from 320 per 1000 people to 377 per 1000 people (17.6%; 95% CI, 14.6%-20.5%; *P* < .001), then decreased to 330 per 1000 people in 2021 (−12.4%; 95% CI, −32.9% to 8.2%; *P* = .59). From 2011 to 2021, ED visits per 1000 people increased by 3.0% (95% CI, −6.7% to 12.7%; *P* = .04), although this increase was not significant.

When examining patterns in ED visits resulting in discharge or hospital admission, the number of ED visits resulting in discharge between 2011 and 2019 increased from 10 133 025 to 12 943 549 (27.7%; 95% CI, 23.7%-31.8%; *P* < .001), while the number of ED visits resulting in admission increased from 1 813 434 to 1 855 365 (2.3%; 95% CI, −0.3% to 5.0%; *P* = .06). From 2019 to 2021, discharged visits decreased from 12 943 549 to 10 963 240 (−15.3%; 95% CI, −37.1% to 6.5%; *P* = .54), while admitted visits increased from 1 855 365 to 1 917 196 (3.3%; 95% CI, −6.0% to 12.6%; *P* = .73). Over the entire study period, discharged visits increased by 8.2% (95% CI, −4.5% to 21.0%; *P* = .67) and admitted visits increased by 5.7% (95% CI, 2.8%-8.7%; *P* = .40), but neither of these increases was significant.

### ED Visits by Acuity

**Figure 2.**
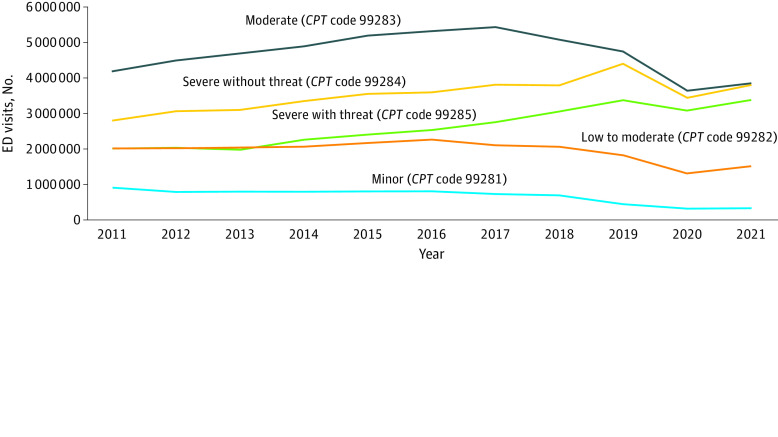
Emergency Department (ED) Visits by Severity *CPT* indicates *Current Procedural Terminology*.

Among all visit acuity groups, the number of visits rated as severe with threat (*CPT* code 99285) increased the most significantly from 2011 to 2021 (from 2 011 637 to 3 375 539; 67.8%; 95% CI, 59.7%-75.9%; *P* < .001), while visits rated as minor (*CPT* code 99281) had the greatest decrease (from 913 712 to 336 071; −63.2%; 95% CI, −75.2% to −51.2%; *P* < .001). The proportion of all visits categorized as severe with threat (*CPT* code 99285) also increased the most significantly over the study period, from 16.7% of visits in 2011 to 26.1% of visits in 2021 (56.3%; 95% CI, 44.9%-67.7%; *P* < .001) (Figure 2). Conversely, the proportion of visits categorized as minor (*CPT* code 99281) had the greatest decrease over the study period (from 7.6% to 2.6%; −65.7%; 95% CI, −81.0% to −50.5%; *P* < .001).

### Ambulance Diversion and Patients Who Left Without Being Seen

During the entire study period, the annual number of ambulance diversion hours increased from 83 558 to 162 217 (94.1%; 95% CI, 62.9%-125.4%; *P* = .09), but there were substantial fluctuations year to year, and this pattern was not statistically significant. The number of patients who left without being seen also fluctuated from 2011 to 2021, and no statistically significant pattern was observed (eg, increase from 303 560 in 2011 to 315 023 in 2021; 3.8%; −4.8% to 12.4%; *P* = .66).

## Discussion

This cohort study found that from 2011 to 2021 in California, the total number of hospitals decreased by 6.2%, the number of hospital beds decreased by 2.5%, and the number of EDs decreased by 3.8%. Over this same period, on aggregate in the entire state, the number of ED visits increased by 7.4%, and the number of ED treatment stations increased by 21.1% in the fewer remaining EDs. There were also significant changes in facility characteristics and visit types; the total number of comprehensive EDs increased by 22.2%, the number of hospitals with TCs increased by 13.8%, and discharged visits increased more than admitted visits (8.2% vs 5.7%, respectively). Visits rated as severe with threat (*CPT* code 99285) increased by 67.8% between 2011 and 2021, while visits rated as minor (*CPT* code 99281) decreased by 63.2%.

Total annual ED visits increased by 23.4%, and total ED visits per 1000 people increased by 17.6% before the onset of the pandemic, revealing that the increase in ED visits exceeded population growth between 2011 and 2019 (5.0%) and that population growth alone could not fully account for the increase in ED visits. This finding follows previous ED visit patterns in California,^21,22^ as the state has had a lower but faster-growing ED visit rate than the national average.^23^ The sharp decrease in visits observed in 2020 and 2021 may be largely associated with the COVID-19 pandemic and closely resembles changes in health care use observed in other states over this same period.^24^

While the total number of ED visits increased, there were also significant changes in visit type. For example, the increase in the number of visits resulting in discharge was larger than that of visits resulting in admission. This finding is consistent with results of a previous study^25^ suggesting a national pattern of decreasing ED admission rates. Some may interpret these findings to mean that the health care system is becoming more organized and EDs are managing patients more efficiently, reducing the need for admissions.^26,27^ Alternatively, and more concerning, is the possibility that there are increasingly stringent criteria for hospital admission given bed scarcity, potentially resulting in patients not receiving the care they need.^28^

The number of visits categorized as severe with threat (*CPT* code 99285) increased more than any other acuity group. At first glance, 1 explanation for this increase could be that ED patients are older and more ill, and there is some evidence to support this claim.^29,30,31^ Alternatively, these changes may have been associated with upcoding, the practice of improperly billing lower-acuity visits as more severe to maximize revenue.^32,33^ There is evidence of upcoding in a previous study,^34^ which found that patient characteristics and services provided only partially explained the generally increasing acuity of ED visits. If an increasing number of lower-acuity patient visits are being upcoded as higher-acuity visits, higher-acuity visit rates will artificially increase.^35,36,37^

Significant changes in capacity may also help explain the observed patterns in use. For example, the total number of hospital beds and hospital beds per 1 million people decreased over the study period. In 2021, California was tied for the 40th lowest state in the nation for its number of hospital beds per 1000 people (3.1 beds per 1000 in California vs 2.4 per 1000 in the US),^38^ and these hospital bed shortages have been identified as a major factor in ED crowding.^39^ Ambulance diversion and patients leaving the ED without being seen can also be symptoms of ED crowding. Ambulance diversion can occur when ED capacity is low and there is a substantial lack of treatment stations or clinicians available. Similarly, patients often leave the ED without being seen due to high waiting times during peak ED crowding.^40^ Although we did not observe significant patterns in these measures, fluctuations may be indicative of ED crowding and should be closely monitored.

Notably, there was a decrease in the number of EDs from 2011 to 2021, which may be a result of facility closures and/or hospital consolidation. Closures of EDs are often a symptom of insufficient hospital funding, and staffing shortages have also been cited as a major challenge in keeping EDs open. With a greater number of sick days and higher rates of burnout among nurses, technicians, and other staff, the strain on EDs has worsened in recent years.^41^

In contrast, the number of ED treatment stations and treatment stations per 1 million people increased over the study period, revealing somewhat conflicting results regarding overall changes in capacity. However, this expansion has happened within a smaller number of total EDs, meaning that certain geographic areas have seen their access to emergency care wane while other areas have seen it expand. A previous study^42^ found that most ED expansion has been localized in affluent (or more commercially insured) areas, supporting the idea that increased ED capacity has not occurred evenly across all populations.

Facility characteristics should also be considered when examining changes in use. While the overall number of EDs decreased over the study period, the number of comprehensive EDs increased and the number of standby and basic EDs decreased, consistent with findings from a previous study^43^ suggesting a disproportionate number of closures for standby EDs. Alternatively, existing EDs may have shifted to higher levels of service, indicating a growing number of facilities that are better equipped to address a broad range of patient conditions. Comprehensive EDs provide the most extensive scope of services, and both basic and comprehensive EDs have a strict nurse to patient ratio of 1 to 4.^44^

The number of hospitals with TCs also increased over the study period in accordance with previous literature,^45,46^ with the greatest growth in lower-level TCs. In 2002, 90% of level I TCs were operating at or above capacity, largely due to financial constraints^47,48^; since then, coordinated efforts have been made to increase TC planning across California.^47,49,50,51^

When examining facilities by ownership, we found that not-for-profit hospitals were consistently the most common type of hospital in California, while the number of government-owned hospitals decreased over time. This finding is consistent with results from a study of previous patterns in ownership,^52^ which found that nationally, public hospitals have closed at a faster rate than private hospitals, mainly due to the government’s financial constraints after the 2008 recession. This pattern is particularly concerning because public hospitals are major sources of safety net care, and closures have been associated with decreased access to care and worse overall health among patients in surrounding communities.^52,53,54^

### Limitations

This study has several limitations. First, we only analyzed patterns in California, which has a distinct health care system and differs from other states in its adoption of policies related to health care access.^55^ Because of these differences, our findings may not be generalizable to all hospitals in the US. Second, a proportion of the changes in the number of hospitals, EDs, and TCs may have been due to mergers rather than ED closures. Nationwide, mergers occur with a frequency that is similar to closures, and there is evidence that studies that do not account for mergers overestimate the number of ED closures by up to a 78% error rate.^27^ Because the data we used did not differentiate between closures and mergers, our study was unable to account for the difference between them. Third, ED treatment stations might not always represent available ED beds (ie, staffed beds) because staffing shortages and other factors may lower actual capacity. For this reason, our study includes various measures of ED capacity. Fourth, our study did not account for frequent users of the ED, which could produce inflated ED visit rates; however, findings from a previous study^56^ suggest that frequent users comprise only 21% to 28% of all ED visits, so we would not expect this factor to substantially alter our results.

## Conclusions

The findings of this cohort study largely suggest that changes in ED capacity have not kept pace with population growth or actual ED use. The COVID-19 pandemic, during which ED use generally decreased, shifted some of these ongoing patterns. To efficiently and equitability address ED crowding and improve overall ED care, policy makers and health care administrators should work toward not only increasing ED capacity, but also making thoughtful decisions about where and how to best allocate resources.

## Notes

Supplement.
Data Sharing Statement
