# Supplementary material for: Patterns in Patient Encounters and Emergency Department Capacity in California, 2011-2021
Source: JAMA Netw Open. 2023 Jun 22;6(6):e2319438. doi: 10.1001/jamanetworkopen.2023.19438 (PMC10288334; doi:10.1001/jamanetworkopen.2023.19438)
Supplement: Supplement. — Data Sharing Statement [file jamanetwopen-e2319438-s001.pdf]

Data Sharing Statement

Hsia. Patterns in Patient Encounters and Emergency Department Capacity in California, 2011-2021. *JAMA Netw Open*. Published June 22, 2023. doi:10.1001/jamanetworkopen.2023.19438

Data
Data available: No
